# N‐Terminomics for the Identification of In Vitro Substrates and Cleavage Site Specificity of the SARS‐CoV‐2 Main Protease

**DOI:** 10.1002/pmic.202000246

**Published:** 2020-11-17

**Authors:** Tomas Koudelka, Juliane Boger, Alessandra Henkel, Robert Schönherr, Stefanie Krantz, Sabine Fuchs, Estefanía Rodríguez, Lars Redecke, Andreas Tholey

**Affiliations:** ^1^ Systematic Proteome Research and Bioanalytics Institute for Experimental Medicine, Christian‐Albrechts‐Universität zu Kiel Kiel 24105 Germany; ^2^ Institute of Biochemistry University of Luebeck Luebeck 23562 Germany; ^3^ Photon Science Deutsches Elektronen Synchrotron (DESY) Hamburg 22607 Germany; ^4^ Experimental Trauma Surgery, Department of Trauma Surgery and Orthopedics University Medical Center Schleswig‐Holstein Kiel 24105 Germany; ^5^ Virology Department Bernhard Nocht Institute for Tropical Medicine German Center for Infection Research (DZIF), Partner site Hamburg‐Lübeck‐Borstel‐Riems, Hamburg, Germany Hamburg 20359 Germany

**Keywords:** Covid19, isobaric labeling, LC‐MS, protease substrates, terminomics

## Abstract

The genome of coronaviruses, including SARS‐CoV‐2, encodes for two proteases, a papain like (PL^pro^) protease and the so‐called main protease (M^pro^), a chymotrypsin‐like cysteine protease, also named 3CL^pro^ or non‐structural protein 5 (nsp5). M^pro^ is activated by autoproteolysis and is the main protease responsible for cutting the viral polyprotein into functional units. Aside from this, it is described that M^pro^ proteases are also capable of processing host proteins, including those involved in the host innate immune response. To identify substrates of the three main proteases from SARS‐CoV, SARS‐CoV‐2, and hCoV‐NL63 coronviruses, an LC‐MS based N‐terminomics in vitro analysis is performed using recombinantly expressed proteases and lung epithelial and endothelial cell lysates as substrate pools. For SARS‐CoV‐2 M^pro^, 445 cleavage events from more than 300 proteins are identified, while 151 and 331 M^pro^ derived cleavage events are identified for SARS‐CoV and hCoV‐NL63, respectively. These data enable to better understand the cleavage site specificity of the viral proteases and will help to identify novel substrates in vivo. All data are available via ProteomeXchange with identifier PXD021406.

Coronaviruses (CoVs) have been identified to cause several human diseases associated with symptoms that range from mild, such as the common cold, to more severe and lethal syndromes such as the severe acute respiratory syndrome (SARS), Middle East Respiratory syndrome (MERS), and the on‐going coronavirus disease 2019 (COVID19). COVID19 appeared in December 2019 in Wuhan, China, and it is caused by a newly identified CoV, SARS‐CoV‐2. This virus led to an initial local outbreak of the disease, followed by a pandemic spread^[^ ^1^ ^]^ affecting more than 28 million people and causing more than 900 000 deaths worldwide (as of September 11, 2020, https://covid19.who.int).

Coronaviruses are enveloped, positive strand RNA viruses and have the largest genome (26–32 kb) of all known RNA viruses.^[^ ^2^ ^]^ The 5’‐terminal end of the genome contains the two open‐reading frames (ORFs) 1a and 1b. The first ORF encodes polyprotein 1a while ORF1a and ORF1b together encode polyprotein 1ab, a mechanism that features a (−1) ribosomal frameshift overreading the stop codon of ORF1a, from which 16 functional proteins are formed by proteolytic processing.^[^ ^2^ ^]^

The proteolytic processing of the translated polyprotein is catalyzed by two types of viral proteases, a papain‐like cysteine protease (PL^pro^) and a chymotrypsin‐like cysteine protease, also called 3C‐like protease (3CL^pro^), main protease (M^pro^) or non‐structural protein 5 (nsp5).^[^ ^2^ ^]^ M^pro^ is responsible for at least ten cleavages along the viral polyprotein, preferably hydrolysing the peptide bonds C‐terminal to glutamine residues within the sequence motif (small amino acid)‐X‐(L/F/M)‐Q↓(G/A/S)‐X (where X is any amino acid; ↓ cleavage site).^[^ ^3^ ^]^ The cleavage of the viral polyprotein produces the mature non‐structural proteins (nsp), which then form the replication/transcription complex.^[^ ^3^ ^]^ In addition, the M^pro^ of SARS‐CoV and the M^pro^ of other coronaviruses has also been shown to counteract the host innate immune response.^[^ ^2^ ^]^ For example, the M^pro^ of the porcine deltacoronavirus (PDCoV) cleaves NF‐κB essential modulator (NEMO) thereby inhibiting interferon‐β production (IFN‐β) and downstream signalling.^[^ ^4^ ^]^ Human A549 and HuH7 cells infected with human coronavirus 229E also showed degradation of NEMO.^[^ ^5^ ^]^ Furthermore, the JAK‐STAT pathway is impaired by processing porcine STAT2, thereby reducing interferon‐stimulated gene (ISG) expression.^[^ ^6^ ^]^

Most of the known substrates of CoV M^pro^ have been identified by targeted biochemical analyses or were predicted using consensus cleavage sites.^[^ ^7^ ^]^ However, bioinformatics and candidate approaches are hypothesis driven and may not fully capture physiologically relevant protease substrates. Mass spectrometry‐based methods have been successfully utilized to identify substrates of the M^pro^ from poliovirus and coxsackievirus B3, members of the *Enterovirus* genus in the picornavirus family.^[^ ^8^ ^]^ Here, protease‐generated N‐termini were enriched via negative selection using an approach named terminal amine isotopic labeling of substrates (TAILS), followed by identification of the corresponding peptides by tandem mass spectrometry.^[^ ^9^ ^]^ The advantage of the TAILS approach is that the detection of a cleaved peptide by MS simultaneously identifies the substrate and the corresponding cleavage site. Using this unbiased proteomics approach, Jagdeo and colleagues identified about 100 novel host targets of the enterovirus 3C protease.^[^ ^8^ ^]^ In the present study, we employed a modified TAILS approach to identify substrates of the M^pro^ from three coronaviruses: CoV‐NL63 (hCoV‐NL63), SARS‐CoV, and SARS‐CoV‐2, by incubating the different proteases with lysates from different lung cells.

Cell lysates from lung epithelial carcinoma cells (H441) and human pulmonary microvascular endothelial cells (HPMEC)^[^ ^10^ ^]^ were incubated with and without the different M^pro^ in triplicate. Subsequently, samples were labeled with TMT 6‐plex using standard protocols and digested with trypsin. Neo N‐termini generated by trypsin were depleted using hydrophobic tagging‐assisted N‑termini enrichment (HYTANE).^[^ ^11^ ^]^ The samples were analyzed in duplicate via reversed‐phase nano liquid chromatography coupled online to either a Q Exactive MS or an Orbitrap Fusion Lumos Tribrid MS (Thermo Fisher Scientific, Bremen, Germany). The latter was additionally equipped with high‐field asymmetric‐waveform ion‐mobility spectrometry (FAIMS). Samples were measured in duplicate on the Q Exactive. For the Lumos, two injections were also performed but run at slightly different FAIMS compensation voltages (CV), that is, −40, −60, −75 CV, and −45, −65, −85 CV, to identify complementary peptides.

The final dataset consisting of 24 files was analyzed with Proteome Discoverer using the SequestHT search algorithm. High‐confidence peptides were filtered for N‐termini containing a TMT‐tag and these were evaluated using the Perseus (1.6.10.43) software package. N‐termini were filtered by log_2_ fold‐change and an acceptable q‐value (0.05). Cell culture, protein expression, methodologies, LC‐MS details, database searching parameters, and data evaluation are provided in the Supporting Information.

For each cell line and M^pro^, samples were measured on both the Q Exactive and the Orbitrap Fusion Lumos Tribrid MS with FAIMS attached. N‐termini analyses led to the identification of approximately 1600–2000 proteins, 3800–6000 peptides, and 50–260 high‐confidence cleavage events (Table S1, Supporting Information). In comparison to results achieved on the Q Exactive MS, measurements utilizing FAIMS resulted in a 22% to 36% increase in the number of peptides identified in the H441 and HPMEC lysates after SARS‐CoV‐2 M^pro^ treatment, respectively (Tables S1 and S2, Supporting Information). More importantly, for the same data set, this resulted in an increase in the number of high‐confidence candidates by 45% and 43% for H441 and HPMEC, respectively (Tables S1 and S2, Supporting Information). While differences in the number of peptide identifications could also be attributed to the increase in acquisition speed of the Fusion Lumos compared to the Q Exactive, the increase in the number of high‐confidence candidates is most likely attributed to the reduction in co‐isolation and ratio suppression by using FAIMS, which has been shown to significantly improve the accuracy and the comprehensiveness of proteomic analyses.^[^ ^12^ ^]^ The use of FAIMS for the hCoV‐NL63 M^pro^‐treated H441 and HPMEC cell lysates increased the number of peptide identifications by 16% and 14%, while the number of high‐confidence candidates increased by 26% and 21%, respectively, compared to the Q Exactive measurements (Tables S1 and S2, Supporting Information). A similar result in terms of the number of peptide identifications and high‐confidence candidates was obtained for the SARS‐CoV M^pro^‐treated cell lysates, regardless of whether they were measured on the Q Exactive or on the Fusion Lumos. This may be due to the low number of cleavage events observed with this particular M^pro^.

In total, we identified 640 high‐confidence cleavage sites corresponding to 434 unique accession numbers (Table S3, Supporting Information). 418 M^pro^‐derived peptides (from 318 unique proteins) had a positive log_2_ change and also contained a glutamine residue at position P1 (Table S3 and Figure S1, Supporting Information). A positive log_2_ fold change identifies peptides that increased in abundance upon M^pro^ addition, indicating that the proteins from which they are part of represent potential M^pro^ substrates. We also detected 75 N‐termini with a negative log_2_ fold change, which may represent natural N‐termini that are degraded upon the addition of M^pro^ (Table S3, Supporting Information). For example, peptide [M].SLKLQASNVTNKNDPKSINSR.[V] from the RNA‐binding protein Raly (RALY) was observed with a log_2_ fold change of −1.28 and −2.12 upon addition of M^pro^ (SARS‐CoV‐2) to H441 and HPMEC lysates, respectively. However, this peptide represents the protein's canonical N‐terminus and also contains the consensus sequence for M^pro^ cleavage (with amino acids LQAS). Therefore, high‐confidence peptides that are found to be degraded upon M^pro^ addition are also of interest, potentially representing protease substrates. Surprisingly, the overlap of the high‐confidence N‐terminal peptides (and their proteins) identified upon addition with the different M^pro^ was very low (Figure S2, Supporting Information), in particular for the main proteases of SARS‐CoV and SARS‐CoV‐2, which are highly similar in terms of their amino acid sequence (96% similarity, Figure S3, Supporting Information) and catalytic efficiency.^[^ ^1^ ^]^ As we observed a slight precipitation of the CoV M^pro^ prior to incubation with the cell lysate, the low number of M^pro^‐derived peptides and the small overlap between SARS‐CoV M^pro^ and SARS‐CoV‐2 M^pro^ derived peptides may be due to the reduced amount or activity of SARS CoV M^pro^.

The cleavage site specificity of proteases is important for substrate prediction and molecular based drug design. The cleavage site specificity of main proteases is deduced from the proteolytic processing of their own viral polyproteins to produce functional nsps.^[^ ^7^, ^13^ ^]^ Indeed, the cleavage information from seven M^pro^s was used to train a neural network and to identify potential novel CoV M^pro^s’ substrates in a human host.^[^ ^7^ ^]^ From these and other analyses, it is clear that M^pro^ prefers the sequence motif (small amino acid)‐X‐(L/F/M)‐Q↓(G/A/S)‐X (where X is any amino acid; ↓ cleavage site) whereby, the glutamine (Q) residue in the P1 position of the substrate is an absolute requirement.^[^ ^3^ ^]^ We compare the cleavage site specificity of the three CoV M^pro^s determined in our in vitro experiments (**Figure** **1**) and compared them to their consensus cleavage site specificity, that is, where they process their own viral polyprotein (Figure S4, Supporting Information). In our in vitro proteomic approach, we identified hundreds of cleavage sites (*n* = 391, 130, and 305, for SARS‐CoV‐2 M^pro^, SARS‐CoV M^pro^, and hCoV‐NL63 M^pro^, respectively) from proteins in their native state and environment, which provides a much more accurate description of the cleavage specificity of the three M^pro^s than those produced in vivo (*n* = 11 for SARS‐CoV and CoV‐2 M^pro^, *n* = 10 for hCoV‐NL63 M^pro^). The cleavage specificity of the individual proteases was similar, with Gln at P1 and Leu at P2 position was consistently required, together with Gly/Ala/Ser at position P1^’^ (Figure 1). At the P4 position, Gly/Ala/Thr were more prevalent, in accordance with previous reports.^[^ ^3^ ^]^ Interestingly, SARS‐CoV‐2 M^pro^ seems to accept a broader variety of amino acids at the P2 position with approximately 59% (230/391) and 6% (23/391) of all M^pro^‐derived peptides exhibiting Leu and Met at the P2 position, respectively. This was previously proposed for M^pro^s from betacoronaviruses like SARS‐CoV and SARS‐CoV‐2 in general, compared to that of alphacoronaviruses.^[^ ^14^ ^]^ Indeed, from all hCoV‐NL63 M^pro^‐derived peptides detected, 73% (221/305) and 4% exhibited leucine and methionine residues at the P2 position, respectively (Figure 1). Next to the low sequence similarity between SARS‐CoV‐2 and hCoV‐NL63 M^pro^ of only 44.3% (Figure S3, Supporting Information), particularly differences in the secondary structure of residues 45 to 51, which form a tight loop in hCoV‐NL63 M^pro^ and a 3_10_ helix in SARS‐CoV M^pro^, are suggested to contribute to these differences.^[^ ^14^ ^]^ The binding of a particular substrate residue at a protease subsite can have either a positive or negative influence on the binding of particular residues at other subsites, a phenomenon coined subsite cooperativity.^[^ ^15^ ^]^ Indeed, it has been shown for SARS‐CoV M^pro^ that a Phe residue at the P2 position leads to a conformational change in the substrate‐binding pocket, creating the subsite for another Phe residue at position P3′.^[^ ^13^ ^]^ This subsite specificity was shown to be required for the C‐terminal autoprocessing of SARS‐CoV M^pro^, which includes a Phe residue at positions P2 and P3’.

**Figure 1:**
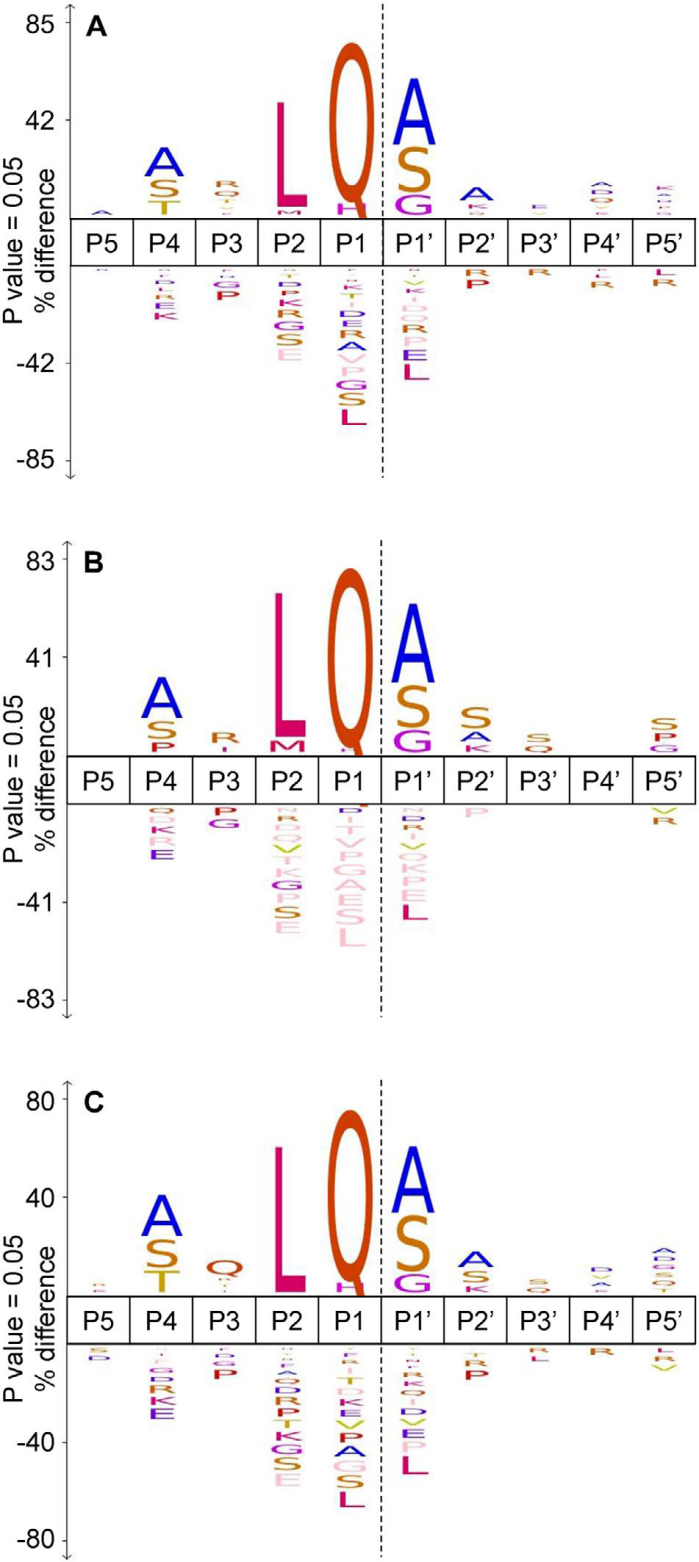
The cleavage site specificity for A) SARS‐CoV‐2 M^pro^ (*n* = 391), B) SARS‐CoV M^pro^ (*n* = 130), and C) hCoV‐NL63 M^pro^ (*n* = 305) derived from in vitro experiments. The precompiled Swiss‐Prot homo sapiens reference set was used as background.

Interestingly, we also observed a significant propensity for the presence of His in the P1 position: with 9.7% (38/391) and 8.5% (26/305) of all protease‐derived peptides exhibiting His in the P1 position for SARS‐CoV‐2 and hCoV‐NL63 M^pro^, respectively. Moreover, the subsite specificity of SARS‐CoV‐2 M^pro^ with His (*n* = 26) at the P1 position was very similar to that with Gln (*n* = 291) at P1 (Figure S5, Supporting Information). Both shared a very similar preference for Leu residues in P2 and Ala and Ser residues in both P4 and P1’, respectively. The lower propensity for His at the P1 position for SARS‐CoV M^pro^ may be due to structural variations in the P1 binding pockets of the different M^pro^s. hCoV‐NL63 M^pro^ forms a smaller S1 pocket than SARS‐CoV M^pro^, optimally harboring the His side chain that is smaller than that of Gln.^[^ ^14^ ^]^ However, this effect might also be attributed to the lower observed proteolytic activity SARS‐CoV M^pro^, compared to SARS‐CoV‐2 and hCoV‐NL63 M^pro^. These results suggest that SARS‐CoV‐2 and hCoV‐NL63 M^pro^, and potentially other CoV M^pro^s, can not only hydrolyse the peptide bond C‐terminal to Gln residues but also His residues, albeit at a lower frequency. This questions the paradigm that Q is essential in the P1 position, potentially enabling the protease to access a wider range of substrates in vivo.

Ultimately, we are interested in novel substrates of M^pro^s to get a better insight into how CoVs interact with the host proteome, for example, to evade the innate immune response. As revealed by our experiments, all three coronavirus M^pro^s were able to cleave NEMO between Q231/V232. NEMO has been previously shown to be cleaved at this position by porcine deltacoronavirus M^pro^ in vivo, validating our in vitro based proteomics approach.^[^ ^4^ ^]^ The M^pro^ from PDCoV, is also able to process porcine STAT2 at positions Q685 and Q758, affecting the structural and functional integrity of STAT2 and subsequent STAT2 phosphorylation and ISG induction.^[^ ^6^ ^]^ STAT2 was not identified in our data set as the amino acids proceeding Q685 are E686 and R687, resulting in a peptide that is too small for mass spectrometry analysis. Furthermore, in human STAT2, Q758 is replaced by a Leu residue, perturbing M^pro^s ability to cleave at this position.

M^pro^ from hCoV‐NL63 and SARS‐CoV‐2 were able to cleave optineurin (OPTN) at two different sites (LQ151.152AE and LQ165.166LK) (Table S4, Supporting Information). OPTN plays a role in the activation of innate immune response during viral infection. After viral infection and the stimulation of pattern‐recognition receptors, TANK‐binding kinase I (TBK1) is activated by K63‐linked polyubiquitination. The ubiquitin‐binding protein optineurin recruits ubiquitinated TBK1 to the Golgi apparatus, leading to the formation of complexes in which TBK1 is activated by trans‐autophosphorylation.^[^ ^16^ ^]^ Activated TBK1 induces type I interferon production by phosphorylating the transcription factor IRF3. Optineurin deficiency in various cell lines and primary cells impairs TBK1 targeting to the Golgi apparatus and its activation following RLR or TLR3 stimulation.^[^ ^16^ ^]^ Cleavage of OPTN may neutralize its activity and thereby decrease TBK1 activation and its downstream signaling.

Eukaryotic translation initiation factor 4 (EIF4G1) was cleaved at two sites, LQ658.659GI and LQ1127.1128QA, both containing the consensus sequence for M^pro^ cleavage, particularly at the first cleavage site between LQ658.659GI, which also contains a small amino acid at the P1’ position (Table S4, Supporting Information). Cleavage of this protein may lead to host cell shut‐off in a similar way to what has been described for picornavirus 2A proteinase.^[^ ^2^ ^]^

Ubiquitination is important in the regulation of the innate immune response, for example, double‐stranded RNA induce K63‐linked ubiquitination of Retinoic acid‐inducible gene 1 protein (RIG‐1), which facilitates its association with mitochondrial antiviral‐signaling protein (MAVS) for MAVS activation.^[^ ^17^ ^]^ Two E3 ubiquitin ligases, TRIM25 and RIPLET, have been shown to mediate RIG‐I ubiquitination and type I IFN induction.^[^ ^17^ ^]^ Knocking out Riplet abrogates the expression of type‐I IFN in response to Hepatitis C Virus (HCV) RNA.^[^ ^2^ ^]^ Moreover, HCV NS3/NS4A^pro^ can cleave RIPLET and abolish RIG‐1 activation. We identified a large number of E3 ubiquitin ligases that were cleaved by the tested M^pro^s, for examples ITCH, UBE3A, and RNF20 (Table S4, Supporting Information). While these E3 ubiquitin ligases have been shown to be important in other viral infections, for example, UBE3A, RNF20, their importance during CoV infections needs to be further investigated.

Our study gathered significant insight into M^pro^s cleavage site specificity and identified hundreds of novel proteins that may act as substrates for the M^pro^s of SARS‐CoV, SARS‐CoV‐2, and hCoV‐NL63 in vitro. Whether the proteases cleave the same or similar substrates in vivo in the infected host cell needs to be further evaluated. The catalogue of potential substrates will serve as a useful base for future hypothesis‐driven studies.

## Conflict of Interest

The authors declare no conflict of interest.

## Supporting information

Supporting Information

Supporting Information
