# Supplementary material for: N‐Terminomics for the Identification of In Vitro Substrates and Cleavage Site Specificity of the SARS‐CoV‐2 Main Protease
Source: Proteomics. 2020 Nov 17;21(2):2000246. doi: 10.1002/pmic.202000246 (PMC7645863; doi:10.1002/pmic.202000246)
Supplement: Supplementary file 1 — Supporting Information [file PMIC-21-0-s002.docx]

**Supporting Information**

**N-terminomics for the identification of in-vitro substrates and cleavage site specificity of the SARS-CoV-2 main protease**

Tomas Koudelka^1^, Juliane Boger^2^, Alessandra Henkel^2^, Robert Schönherr^2,3^, Stefanie Krantz^4^, Sabine Fuchs^4^, Estefanía Rodríguez^5^, Lars Redecke^2,3^ & Andreas Tholey^1, *^

^1^ Systematic Proteome Research & Bioanalytics, Institute for Experimental Medicine, Christian-Albrechts-Universität zu Kiel, 24105 Kiel, Germany

^2^ Institute of Biochemistry, University of Luebeck, 23562 Luebeck, Germany

^3^ Photon Science, Deutsches Elektronen Synchrotron (DESY), 22607 Hamburg, Germany

^4^ Experimental Trauma Surgery, Department of Trauma Surgery and Orthopedics, University Medical Center Schleswig- Holstein, Kiel, Germany

^5^ Virology Department, Bernhard Nocht Institute for Tropical Medicine, 20359 Hamburg, Germany

**Materials and Methods**

## **Cloning and gene expression**

Cloning of the genes encoding SARS-CoV M^pro^ (GenBank code: AY390556.1) and SARS-CoV-2 M^pro^ (GenBank code MN908947.3) into the pGEX-6p-1 plasmid (GE Healthcare) has been described previously [1,2]. The gene encoding hCoV-NL63 M^pro^ (GenBank code: FJ211861.1) was amplified and also ligated into the pGEX-6p-1 plasmid. The sequence of the generated plasmid was verified by sequencing. For gene expression, YT-medium (1x) containing ampicillin (100 µg/mL) was inoculated with a single colony of *E. coli* BL21-Gold (DE3) cells (Novagen) transformed with the corresponding plasmid and pre-cultured at 37 °C and 180 rpm. After 3 hrs, 2 L 1x YT-medium containing 100 µg/mL ampicillin were seeded with the pre-culture. When the OD_600_ reached 0.8, expression of the SARS-CoV and SARS-CoV-2 M^pro^ gene was induced by adding 0.5 M isopropyl-D-thiogalactoside (IPTG), followed by incubation at 37 °C for 5 hrs. Gene expression of hCoV-NL63 M^pro^ was induced by addition of 0.2 M IPTG at an OD_600_ between 0.8 and 1.0, followed by incubation for 10 hrs at 25 °C. Bacteria were harvested by centrifugation for 15 min at 5,000 rcf and 4 °C. Pellets were resuspended in 1x YT-medium, centrifuged again and stored at -20 °C.

## **Purification of the recombinant proteases**

*E. coli* cell pellets were thawed on ice, resuspended in 30 mL buffer A (20 mM Tris, 150 mM NaCl, pH 7.8) and cells were lysed using sonication on ice. The obtained lysate was ultracentrifuged at 146,682 rcf for 1 hr at 4 °C. For purification of SARS-CoV and SARS-CoV-2 M^pro^ a His-Trap FF column (GE Healthcare) equilibrated with buffer A was loaded with the cleared lysate and washed using 150 mL buffer A, while 4 % buffer B (20 mM Tris, 150 mM NaCl, 500 mM imidazole, pH 7.8) in buffer A was used for the washing step in terms of hCoV-NL63 M^pro^. Elution was performed using a linear gradient of buffer B ranging from 0 % to 100 % in 20 column volumes (CV). The fractions containing the target protein were pooled and the buffer was exchanged.

Pooled fractions of SARS-CoV-2 M^pro^ were dialysed into buffer C (20 mM Tris, 150 mM NaCl, 1 mM DTT, pH 7.8) using a molecular weight cut off of 10 kDa overnight at 4 °C, followed by an exchange into buffer D (20 mM Tris, 1 mM DTT, pH 8.0) using Amicon Ultra centrifugal filters (10 kDa) at 4,000 rcf. Pooled fractions of SARS-CoV M^pro^ were only subject to buffer exchange into buffer D using the centrifugal filters. The protein was loaded onto a MonoQ column (GE Healthcare) equilibrated with buffer D. The column was washed with 10 CV buffer D. For elution, a linear gradient of buffer E (20 mM Tris, 1 M NaCl, 1 mM DTT, pH 8.0) ranging from 0 % to 100 % in 20 CV was applied. After pooling fractions containing the highly pure target protein, the buffer was exchanged into buffer F (20 mM Tris, 150 mM NaCl, 1 mM EDTA, 1 mM DTT, pH 7.8) and the protein concentrated to 6,8 mg/ml (SARS-CoV) and 3,4 mg/mL (SARS-CoV-2), respectively.

## Elution fractions of the HisTrap FF column containing hCoV-NL63 M^pro^ were pooled, concentrated to a volume of 5 mL and loaded onto a Superdex-200 size exclusion column (GE Healthcare) for further purification. Fractions containing the highly pure target protein were pooled and concentrated to a final concentration of 98 mg/mL.

**Culture of H441 cells**

H441 cells as reference cell line for lung epithelial cells with alveolar type II like cell characteristics were cultured as previously described [3,4] in RPMI 1640 medium (Gibco®/Life Technologies, Paisley, UK) supplemented with 10 % fetal bovine serum (FBS) (Sigma-Aldrich, KS, USA), 1 % penicillin/streptomycin (Biochrom GmbH, Berlin, Germany) and 1 % L-Glutamine (Gibco®/Life Technologies, Paisley, UK) at 37°C with 5 % CO_2_ on cell culture flasks in density of 26.000 cells/cm^2^. After reaching confluency at day 7 H441 cells were detached from the cell culture ware by trypsinization using standard procedures. Biological replicates were generated by cells from different passage numbers. After washing in PBS for serval times cells were kept on ice prior to immediate cell lysis or frozen at -80°C for later use.

**Isolation of microvascular endothelial cells from human lung tissues and harvesting for the treatment with SARS-COV enzymes**

The isolation of cells from human tissue was performed with the consent from the local ethical advisory board and from individual patients undergoing lung tumor resections at the UKSH in Kiel. The isolation and characterization of microvascular endothelial cells was performed as previously published in detail [5,6]. In brief, human lung tissue was dissected into small fragments followed by overnight digestion of the tissue in dispase. Pre-digested tissue was further treated with trypsin and elastase for 40 minutes at 37°C to release cells from lung tissue.

After several washing steps the cell suspensions were plated on cell culture flasks for 90 minutes to allow adhesion of endothelial cell populations. The adhesive cells in the cell culture flasks from this panning step were further cultured in endothelial growth medium (EGCM-2, PromoCell (Heidelberg, Germany) supplemented with 7% FBS and 1% Pen/Strep) to expand endothelial cell populations. After reaching sub-confluency endothelial cells were purified by magnetic cell sorting for CD31 (Dynal by Thermofisher, Waltham, USA) resulting in the selection of primary microvascular endothelial cells from human lung (HPMEC). Primary cells were characterized as published earlier [4] for celltype specific markers.

Microvascular pulmonary endothelial cells from different donors in passage 4-6 were seeded on fibronectin coated cell culture flasks in endothelial growth medium as stated above and harvested after reaching confluency. Cells were detached from the cell culture ware by trypsinization using standard procedures. After washing in PBS for serval times cells were kept on ice prior to immediate cell lysis or frozen at -80°C for later use.

***In vitro* N-terminomics**

Cell aliquots (~500,000 cells) were lysed in 100 mM HEPES, 0.15M NaCl (pH 7.4), EDTA (1 mM) and pepstatin (10 µM) using freeze-thaw cycles and sonication in an ice water bath (3 times, 5 min each step, total time 30 min). After centrifugation at 21,100g for 15 min at 4°C, cell lysates were pooled and an aliquot used for protein determination (BCA, Pierce). Lysate was split into 6 aliquots, three aliquots used for control (without M^pro^) and three aliquots were treated with protease and incubated for 16 h (1:10 ratio). Each aliquot contained 50 µg and 25 µg of lysate for H441 and HPMEC, respectively and 2.6 and 2.2 µMol of M^pro^ for H441 and HPMEC, respectively. Samples were incubated with relatively high amounts of protease:protein ratio to ensure that the protease was present as an active dimer (2 µmolL^-1^). Each sample was then precipitated to quench the reaction and to remove amino acids and cellular material that may negatively impact TMT labelling. Individual sample pellets were resuspended in 6M Guanidine-hydrochloride, 100 mM TEAB (triethyl-ammonium bicarbonate) with the addition of 5 mM Tris(2-carboxyethyl)phosphine hydrochloride (TCEP). The samples were heated (95 °C) for 10 min and then left to reduce for an additional 30 min at 56 °C. Samples were alkylated with 12.5 mM iodoacetamide at 25 °C in the dark for 30 min. Proteins were then labelled with the particular TMT-reagent (TMT-6-plex) in a final DMSO concentration of 50% for 1h at 25 °C before being quenched with 8 µL of 10% hydroxylamine for 30 min at 37 °C. Samples were combined and methanol/chloroform/water precipitated. The pellet was washed with methanol, air-dried for 15 min and then resuspended in 3 M guanidine-hydrochloride in 100 mM TEAB. Samples were digested with trypsin at 50:1 (protein:enzyme) for 16 h at a final guanidine-hydrochloride concentration of <1M at 37 °C. Peptides were cleaned (C-18, Sep-Pak, Waters) and 10% of the sample used for “PRE-HYTANE”, the rest was used to deplete internal peptides using the HYTANE strategy. While classical TAILS encompasses large dendritic polyglycerol aldehyde for negative selection of internal peptides, the HYTANE strategy uses a hydrophobic aldehyde to bind to trypsin-derived neo-N-termini, which is later on removed. HYTANE strategy was performed as described previously with minor modifications [7]. Briefly, for H441 samples peptides (~270 µg) were resuspended in 150 µL of HEPES buffer (200 mM, pH 7.0) and incubated with 500 µL of hexadecanal dissolved in isopropanol (10 mg/mL). Sodium cyanoborohydride (NaCNBH_3_, 18 mM final conc.) was added at left to incubate for approximately 3 hours at 50°C followed by overnight incubation at 37°C. A fresh aliquot of NaCNBH_3_ was added and the sample dried by vacuum centrifugation for 3 h. For HPMEC samples (~135 µg) all volumes were halved. Samples were acidified with 10% TFA and made up to 1 mL with HPLC loading buffer (0.1% TFA, 3% acetonitrile (ACN)). Sample was spun down at 21,100g for 10 min and cleaned via C-18 Sep-Pak (50 mg, 1cc, Waters). Samples were dried (vacuum evaporation) and stored at -20°C prior to analysis.

10-20 µg (equivalent of starting material) was injected in duplicate on a Ultimate 3000 nano-UHPLC coupled to a Q Exactive mass spectrometer or an Orbitrap Fusion Lumos Tribrid mass spectrometer (both Thermo Scientific, Bremen, Germany). The samples were washed on a trap column (Acclaim Pepmap 100 C-18, 5 mm × 300 μm, 5 μm, 100 Å, Thermo Scientific) for 4 min with 3% ACN/0.05% TFA at a flow rate of 30 μl/min prior to peptide separation using an Acclaim PepMap 100 C-18 analytical column (50 cm × 75 μm, 2 μm, 100 Å, Thermo Scientific). A flow rate of 300 nL/min using eluent A (0.05% FA) and eluent B (80% ACN/0.04% FA) was used for gradient separation (180-minute gradient, 5-40% B).

Analyses performed on the Q Exactive MS: the spray voltage applied on a metal-coated PicoTip emitter (10 μm tip size, New Objective, Woburn, Massachusetts, US) was 1.6-1.7 kV, with a source temperature set to 250°C. Full scan MS spectra were acquired between 300 and 2,000 m/z at a resolution of 70,000 at m/z 200. The ten most intense precursors with charge states greater than 2+ were selected with an isolation window of 1.4 m/z and fragmented by HCD with normalized collision energies of 33 at a resolution of 17,500. Lock mass (445.120025) and dynamic exclusion (30 seconds) were enabled.

Measurements performed on the Fusion Lumos MS, with FAIMS attached: the spray voltage applied was 1.8-1.95 kV, with a source temperature set to 300°C. Full scan MS spectra were acquired between 375 and 1,400 m/z at a resolution of 60,000. The most intense precursors within 3 seconds (1 sec/CV) with charge states greater than 2+ were selected, with an isolation window of 1.2 m/z, and fragmented by HCD with normalized collision energies of 40 at a resolution of 30,000. A dynamic exclusion of 30 seconds was enabled. Two injections were performed but scanned at different compensation voltages to try and identify complementary peptides, i.e., -40, -60, -75 CV and -45, -65, -85 CV. FAIMS resolution mode was set to standard. FAIMS electrodes were operated at a dispersion voltage (DV) of −5000 V using a 5.0 L/min flow of nitrogen (cooling gas).

**Database Search and Statistics**

The MS raw files were processed by Proteome Discoverer 2.2 (2.2.0.388) and 2.4 (2.4.1.15) and MS/MS spectra were searched using the Sequest HT algorithm against a database containing the canonical and reviewed human proteome (February 2020 release data, <https://www.uniprot.org/>, 20,364 entries) with the common contaminants (<https://www.thegpm.org/crap/>, 47 entries), *Escherichia coli* (strain B/BL21-DE3, 4,156 entries), M^pro^ from hCoV-NL63, SARS-CoV and SARS-CoV-2 appended to the database. Percolator (v3.0) was used for error calculation with a peptide level false discovery rate restricted to <0.01. The enzyme specificity was set to semi-ArgC with three missed cleavages allowed. An MS1 tolerance of 10 ppm (15 ppm for FAIMS) and a MS2 tolerance of 0.02 Da was implemented. A larger MS1 mass tolerance was allowed for FAIMS data because no lock mass is possible during acquisition.

Oxidation (15.995 Da) of methionine residues, acetylation (42.011 Da) and TMT-6-plex (229.163 Da) on the peptide N-terminus was set as a variable modification while carbamidomethylation (57.02146 Da) on cysteine residues and TMT-6-plex on lysine residues was set as a static modification. Technical injection replicates were set as fractions.

Normalized and scaled abundance from Proteome Discoverer were exported to Microsoft Excel, log_2_ transformed and filtered for N-termini and quan. value. Peptides containing an N-acetylation and a TMT-tag (on lysine residues) were also retained. Peptides were exported to Perseus (version 1.6.10.43) and further filtered: for analyses performed on the Q Exactive only peptides with values in 2 from 3 TMT channels per group (with and without protease) were included, while for the Fusion Lumos data 3 from 3 TMT channels had to be present in at least one group. Here missing channels were imputed (a normal distribution downshifted from the channel mean) as co-isolation was significantly reduced with the addition of FAIMS. Nevertheless, only a very small number of peptides were filtered using the above criteria. A t-test was performed comparing control and protease treated samples and to compensate for the multiple testing hypothesis, a permutation based FDR value of 0.05 (5%) was also performed. Aside from an acceptable q-value, only peptides which exhibited log_2_ fold changes less/greater than 2 SD from the sample median were considered. Peptides belonging to the M^pro^, common contaminants, and *E.coli* were also removed. For cleavage site specificity, peptides were filtered to only include peptides with a positive log_2_ fold change, i.e., more abundant upon addition of protease, and peptides which did not start at the first or second position within a protein i.e., protein N-terminus. The cleavage consensus sequence for the different M^pro^s was performed using the program iceLogo (<https://iomics.ugent.be/icelogoserver/>) [8].

**Supporting Figures and Tables**

**Supplementary Table 1**: Summary of the LC-MS results from different proteases and cell lines. ^1^ HC-Cleavage events: peptides that were found to be statistically different (p-value adjusted) between M^pro^ and control and with a log_2_ fold change more than 2 standard deviations away from the sample median. Peptides that belonged to *E.coli* and the proteases themselves were filtered at this stage. ^2^ HC-Cleavage events that were found to be more abundant in protease treated sample (i.e, a positive log_2_ fold change) and which didn’t start at either position 1 or 2. “Cleavage sites” were analyzed by the program iceLogo (<https://iomics.ugent.be/icelogoserver/>) to determine M^pro^s’ cleavage site specificity.

| Main  Protease | Cell line | Instrument | Proteins | Peptides | N-term Pep. | N-term Pep. (TMT or Acetyl+TMT) | HC-Cleavage events^1^ | Cleavage events | Cleavage sites^2^ |
| --- | --- | --- | --- | --- | --- | --- | --- | --- | --- |
| SARS-CoV-2 | H441 | Q Excactive | 2045 | 5056 | 4590 | 3926 | 179 | 445 | 391 |
|  |  | Lumos  (FAIMS) | 2169 | 6149 | 5346 | 4759 | 260 |  |  |
|  | HPMEC | Q Excactive | 1723 | 3842 | 3530 | 2977 | 149 |  |  |
|  |  | Lumos  (FAIMS) | 1921 | 5237 | 4581 | 4060 | 213 |  |  |
| SARS-CoV | H441 | Q Excactive | 1735 | 4292 | 3372 | 2774 | 59 | 151 | 130 |
|  |  | Lumos  (FAIMS) | 1652 | 4302 | 3261 | 2783 | 52 |  |  |
|  | HPMEC | Q Excactive | 1726 | 4426 | 3334 | 2711 | 71 |  |  |
|  |  | Lumos  (FAIMS) | 1910 | 5816 | 4237 | 3623 | 83 |  |  |
| hCoV-NL63 | H441 | Q Excactive | 1670 | 4083 | 3178 | 2648 | 125 | 331 | 305 |
|  |  | Lumos  (FAIMS) | 1765 | 4737 | 3504 | 3028 | 158 |  |  |
|  | HPMEC | Q Excactive | 1681 | 4116 | 3129 | 2545 | 132 |  |  |
|  |  | Lumos  (FAIMS) | 1737 | 4676 | 3404 | 2884 | 160 |  |  |

**Supplementary Table 2**. Comparison of the LC-MS results between the Q Exactive and the Fusion Lumos/FAIMS. Values shown represent the percentage differences between the two instruments shown in **Supplementary Table 1**. ^1^ HC-Cleavage events: peptides that were found to be statistically different (p-value adjusted) between protease and control and with a log_2_ fold change more than 2 standard deviations distance from the sample median. Peptides that belonged to *E.coli* and the proteases themselves were filtered at this stage.

| Main  Protease | Cell line | Proteins | Peptides | N-term Pep. | N-term Pep. (TMT or Acetyl+TMT) | HC-Cleavage events^1^ |
| --- | --- | --- | --- | --- | --- | --- |
| SARS-CoV-2 | H441 | 106.1% | 121.6% | 116.5% | 121.2% | 145.3% |
|  | HPMEC | 111.5% | 136.3% | 129.8% | 136.4% | 143.0% |
| SARS-CoV | H441 | 95.2% | 100.2% | 96.7% | 100.3% | 88.1% |
|  | HPMEC | 110.7% | 131.4% | 127.1% | 133.6% | 116.9% |
| hCoV-NL63 | H441 | 105.7% | 116.0% | 110.3% | 114.4% | 126.4% |
|  | HPMEC | 103.3% | 113.6% | 108.8% | 113.3% | 121.2% |

**Supplementary Table 3**: see separate Excel sheet

**Supplementary Table 4**: Selection of known and possible protein substrates and their cleavage sites. Log_2_ fold change from the different M^pro^s are shown.

| Gene Name | Description | P4-P1 | Annotated Sequence | SARS-CoV-2 | | | | hCoV-NL63 | | | | SARS-CoV | | | |
| --- | --- | --- | --- | --- | --- | --- | --- | --- | --- | --- | --- | --- | --- | --- | --- |
|  |  |  |  | H441 FAIMS | H441 QExac. | HPMEC  FAIMS | HPMEC QExac. | H441  FAIMS | H441  QExac. | HPMEC  FAIMS | HPMEC  QExac. | H441 FAIMS | H441 QExact. | HPMEC FAIMS | HPMEC QExact. |
| EIF4G1 | Eukaryotic translation initiation factor 4 gamma 1 | TRLQ | [Q].GINCGPDFTPSFANLGR.[T] |  |  |  |  |  | 1.73 |  | 1.46 | 1.33 | 1.75 |  |  |
|  |  | SALQ | [Q].QAVPTESTDNR.[R] |  |  |  |  |  | 3.01 |  | 2.10 |  |  |  |  |
|  |  | SALQ | [Q].QAVPTESTDNRR.[V] |  |  |  |  |  | 1.44 |  |  |  |  |  |  |
| NEMO | NF-kappa-B essential modulator | AQLQ | [Q].VAYHQLFQEYDNHIKSSVVGSER.[K] | 1.42 |  | 1.32 |  | 2.11 |  | 1.84 |  |  |  | 1.44 |  |
| OPTN | Optineurin | VRLQ | [Q].AEKADLLGIVSELQLKLNSSGSSEDSFVEIR.[M] |  |  |  |  |  |  | 1.36 |  |  |  |  |  |
|  |  | SELQ | [Q].LKLNSSGSSEDSFVEIR.[M] |  |  | 3.19 | 1.40 |  |  |  |  |  |  |  |  |
| ITCH | E3 ubiquitin-protein ligase Itchy homolog | ATSQ | [Q].SKEFDPLGPLPPGWEKR.[T] | 1.41 |  |  |  |  |  |  |  |  |  |  |  |
| RNF20 | E3 ubiquitin-protein ligase BRE1A | SKLQ | [Q].SKVETAESR.[V] |  |  | 3.28 | 1.74 |  | 1.76 |  |  |  |  |  |  |
| UBE3A | Ubiquitin-protein ligase E3A | SLVQ | [Q].GQQLNPYLR.[L] |  |  |  | 1.38 |  |  |  |  |  |  |  |  |

**Supplementary Figure 1**. Volcano plots generated to recognize high-confident cleavage events. Log_2_ fold changes and q-values (representing and adjusted p-value or FDR of 5%) are shown for the different M^pro^s and with the different cell lysates (H441 and HPMEC).

**Supplementary Figure 1** continued.

**Supplementary Figure 2**: Overlap of the proteins (**A**) and peptides (**B**) identified in H441 and HPMEC upon incubation with M^pro^ for SARS-CoV, SARS-CoV-2 and hCoV-NL63.

**Supplementary Figure 3**: Sequence alignment of the three M^pro^s using (<https://www.uniprot.org/align>). Sequence identity of all three M^pro^s was 42.7% (131 identical positions), while the sequence alignment between SARS-CoV M^pro^ and SARS-CoV-2 M^pro^ was 96.1% (294 identical positions). The M^pro^ sequence alignment of SARS-CoV-2 and hCoV-NL63 was 44.3% (136 identical positions).

**Supplementary Figure 4**. Canonical cleavage site specificity deribed form the proteolytis of the viral polyprotein, for SARS-CoV-2 M^pro^ (n=11), SARS-CoV (n=11) and hCoV-NL63 (n=10) analyzed by the program iceLogo (<https://iomics.ugent.be/icelogoserver/>) with precompiled Swiss-Prot Human SARS and Human CoV-NL63 reference sets as background.

**Supplementary Figure 5**. SARS-CoV-2 subsite specificity with (**A**) Histidine (n=36) and (**B**) Glutamine (n=291) in position P1 analyzed by the program iceLogo (<https://iomics.ugent.be/icelogoserver/>) with the precompiled Swiss-Prot *homo sapiens* reference sets used as background.

**References**

[1] X. Xue, H. Yang, W. Shen, Q. Zhao, J. Li, K. Yang, C. Chen, Y. Jin, M. Bartlam, Z. Rao, *J. Mol. Biol.* **2007**, *366* (3), 965–975. DOI: 10.1016/j.jmb.2006.11.073.

[2] L. Zhang, D. Lin, X. Sun, U. Curth, C. Drosten, L. Sauerhering, S. Becker, K. Rox, R. Hilgenfeld, *Science*. **2020**, *368* (6489), 409. DOI: 10.1126/science.abb3405.

[3] H. Janga, L. Cassidy, F. Wang, D. Spengler, S. Oestern-Fitschen, M. F. Krause, A. Seekamp, A. Tholey, S. Fuchs, *J. Cell. Mol. Med.* **2018**, *22* (2), 982–998. DOI: 10.1111/jcmm.13421.

[4] D. Spengler, S. Winoto-Morbach, S. Kupsch, C. Vock, K. Blöchle, S. Frank, N. Rintz, M. Diekötter, H. Janga, M. Weckmann, et al., *Am. J. Physiol. Lung Cell Mol. Physiol.* **2018**, *314* (1), L32–L53. DOI: 10.1152/ajplung.00128.2017.

[5] S. Fuchs, A. Hollins, M. Laue, U. Schaefer, K. Roemer, M. Gumbleton, C.-M. Lehr, *Cell Tissue Res*. **2003**, *311* (1), 31–45. DOI: 10.1007/s00441-002-0653-5.

[6] M. I. Hermanns, S. Fuchs, M. Bock, K. Wenzel, E. Mayer, K. Kehe, F. Bittinger, C. J. Kirkpatrick, *Cell Tissue Res.* **2009**, *336* (1), 91–105. DOI: 10.1007/s00441-008-0750-1.

[7] L. Chen, Y. Shan, Y. Weng, Z. Sui, X. Zhang, Z. Liang, L. Zhang, Y. Zhang, *Anal. Chem.* **2016**, *88* (17), 8390–8395. DOI: 10.1021/acs.analchem.6b02453.

[8] N. Colaert, K. Helsens, L. Martens, J. Vandekerckhove, K. Gevaert, *Nat. Methods*. **2009**, *6* (11), 786–787. DOI: 10.1038/nmeth1109-786.
